# Joint EANM/EANO/RANO/SNMMI practice guideline/procedure standard for PET imaging of brain metastases: version 1.0

**DOI:** 10.1007/s00259-024-07038-5

**Published:** 2025-01-07

**Authors:** Antoine Verger, Nelleke Tolboom, Francesco Cicone, Susan M. Chang, Julia Furtner, Norbert Galldiks, Jens Gempt, Eric Guedj, Raymond Y. Huang, Derek R. Johnson, Ian Law, Emilie Le Rhun, Susan C. Short, M. J. Van den Bent, Donatienne Van Weehaeghe, Michael A. Vogelbaum, Patrick Y. Wen, Nathalie L. Albert, Matthias Preusser

**Affiliations:** 1 Department of Nuclear Medicine & Nancyclotep Imaging Platform, CHRU Nancy and IADI INSERM, UMR 1254, Université de Lorraine, Nancy, France; 2 Department of Radiology and Nuclear Medicine, University Medical Centre Utrecht, Utrecht, The Netherlands; 3 Nuclear Medicine Unit, Department of Experimental and Clinical Medicine, “Magna Graecia” University of Catanzaro, Catanzaro, Italy; 4 Division of NeuroOncology, Department of Neurological Surgery, University of California San Francisco (UCSF), San Francisco, CA USA; 5 Research Center for Medical Image Analysis and Artificial Intelligence (MIAAI), Faculty of Medicine and Dentistry, Danube Private University, Krems, 3500 Austria; 6 Department of Neurology, Medical Faculty and University Hospital of Cologne, Institute of Neuroscience and Medicine (INM-3), Research Center Juelich, University of Cologne, Juelich, Germany; 7 Department of Neurosurgery, University Medical Center Hamburg-Eppendorf, Hamburg, Germany; 8 Département de Médecine Nucléaire, Hôpital de la Timone, CERIMED, Institut Fresnel, Aix Marseille University, APHM, CNRS, Centrale Marseille, Marseille, France; 9 Department of Radiology, Brigham and Women’s Hospital, Harvard Medical School, Boston, MA USA; 10 Department of Radiology, Mayo Clinic, Rochester, MN USA; 11 Department of Clinical Physiology and Nuclear Medicine, Rigshospitalet, Copenhagen, Denmark; 12 Departments of Neurosurgery and Neurology, University Hospital Zurich, Zurich, Switzerland; 13 Leeds Institute of Medical Research at St James’s, University of Leeds, Leeds, UK; 14 Department of Neurology, Brain Tumor Center at Erasmus MC Cancer Institute, University Medical Center Rotterdam, Rotterdam, Netherlands; 15 Department of Radiology and Nuclear Medicine, Ghent University Hospital, C. Heymanslaan 10, Ghent, 9000 Belgium; 16 Department of NeuroOncology, H. Lee Moffitt Cancer Center and Research Institute, Tampa, FL USA; 17 Center For Neuro-Oncology, Dana-Farber Cancer Institute and Harvard Medical School, Boston, USA; 18 Department of Nuclear Medicine, LMU Hospital, LMU Munich, Munich, Germany; 19 Division of Oncology, Department of Medicine I, Medical University of Vienna, Vienna, Austria

**Keywords:** Brain metastasis, Brain metastases, Metastatic brain tumors, PET, Methionine, FDOPA, FET, Fluciclovine, FDG, Recurrence

## Abstract

This joint practice guideline/procedure standard was collaboratively developed by the European Association of Nuclear Medicine (EANM), the Society of Nuclear Medicine and Molecular Imaging (SNMMI), the European Association of Neuro-Oncology (EANO), and the PET task force of the Response Assessment in Neurooncology Working Group (PET/RANO). Brain metastases are the most common malignant central nervous system (CNS) tumors. PET imaging with radiolabeled amino acids and to lesser extent [^18^F]FDG has gained considerable importance in the assessment of brain metastases, especially for the differential diagnosis between recurrent metastases and treatment-related changes which remains a limitation using conventional MRI. The aim of this guideline is to assist nuclear medicine physicians in recommending, performing, interpreting and reporting the results of brain PET imaging in patients with brain metastases. This practice guideline will define procedure standards for the application of PET imaging in patients with brain metastases in routine practice and clinical trials and will help to harmonize data acquisition and interpretation across centers.

## Preamble

The Society of Nuclear Medicine and Molecular Imaging (SNMMI) is an international scientific and professional organization founded in 1954 to promote the science, technology, and practical application of nuclear medicine. The European Association of Nuclear Medicine (EANM), founded in 1985, is a professional non-profit medical association that facilitates communication worldwide between individuals pursuing clinical and research excellence in nuclear medicine. As of 2023, it comprised 2,900 members. SNMMI and EANM members are physicians, technologists, and scientists specialized in the research and practice of nuclear medicine.

The SNMMI/EANM will periodically define new standards/guidelines for nuclear medicine practice to help advance the science of nuclear medicine and to improve the quality of service to patients. Existing standards/guidelines will be reviewed for revision or renewal, as appropriate, on their fifth anniversary or sooner, if indicated.

Each standard/guideline, representing a policy statement by the SNMMI/EANM, has undergone a thorough consensus process in which it has been subjected to extensive review. The SNMMI and EANM recognize that the safe and effective use of diagnostic nuclear medicine imaging requires specific training, skills, and techniques, as described in each document.

The EANM and SNMMI have written and approved these standards/guidelines to promote the use of nuclear medicine procedures with high quality. These standards/guidelines are intended to assist practitioners in providing appropriate nuclear medicine care for patients. They are not inflexible rules or requirements of practice and are not intended, nor should they be used to establish a legal standard of care. For these reasons and those set forth below, the SNMMI/EANM cautions against the use of these standards/guidelines in litigation in which the clinical decisions of a practitioner are called into question.

The ultimate judgment regarding the propriety of any specific procedure or course of action must be made by medical professionals taking into account the unique circumstances of each case. Thus, there is no implication that an approach differing from the standards/guidelines, standing alone, is below the standard of care. To the contrary, a conscientious practitioner may responsibly adopt a course of action different from that set forth in the standards / guidelines when, in the reasonable judgment of the practitioner, such course of action is indicated by the condition of the patient, limitations of available resources, or advances in knowledge or technology subsequent to publication of the standards/guidelines.

The practice of medicine involves not only the science but also the art of dealing with the prevention, diagnosis, alleviation, and treatment of disease. The variety and complexity of human conditions make it impossible to always reach the most appropriate diagnosis or to predict with certainty a particular response to treatment. Therefore, it should be recognized that adherence to these standards/guidelines will not ensure an accurate diagnosis or a successful outcome, but the practitioner will follow a reasonable and justifiable course of action based on current knowledge, available resources, and the needs of the patient to deliver effective and safe medical care. The sole purpose of these standards/guidelines is to assist practitioners in achieving this objective.

The present guideline/standard was developed collaboratively by the EANM and SNMMI with the European Association of Neuro-Oncology (EANO), and the PET task force of the Response Assessment in Neurooncology Working Group (PET/RANO). It summarizes the views of the Neuroimaging and Oncology committees of the EANM, Brain Imaging Council of the SNMMI, the EANO, and PET/RANO group and reflects recommendations for which the EANM cannot be held responsible. The recommendations should be taken into context of good practice of nuclear medicine and do not substitute for national and international legal or regulatory provisions.

## Introduction

Brain metastases are the most frequent malignant tumors affecting the central nervous system (CNS). They are believed to occur up to ten times more often than primary brain tumors and they occur in around 9–10% of all cancer cases [1, 2]. Lung cancers (40–50% of all metastases), breast cancers (15–20%), and melanomas (5–20%) are the leading primary cancer sources for brain metastases [1]. Their incidence is expected to rise due to prolonged life span, the development of screening programs for brain metastases in cancer patients, and with novel therapeutics available for cancer. However, these novel therapeutics present for many agents lower effectiveness inside the CNS than outside due to the blood brain barrier (BBB).

Criteria to guide treatment in brain metastases are no longer uniquely based on primary tumor histology, presence of extracranial metastases, number of sites of brain metastases, clinical presentation or socio-demographical factors, but now include a growing number of relevant molecular alterations [3, 4]. Radiotherapy and surgery are two important treatment pillars for brain metastasis [5]. Over the past decade, stereotactic radiosurgery (SRS) has become standard of care for patients with newly diagnosed brain metastases and good performance status, because of good tumor control and decreased risk of cognitive deficits compared to less targeted radiation techniques. Surgical resection can be proposed in selected cases such as significant mass effect, in cases where no histology is yet available, or in case of a single brain metastasis. Surgical removal of lesions with significant mass effect allows for tapering of the dose of steroids, which is particularly relevant when immunotherapy is considered. Conventional whole-brain radiotherapy is nowadays considered only for patients in whom surgery or SRS is not recommended, for example, in patients with several large brain metastases, yet with a survival expectancy of at least several months. The most common late complication of stereotactic irradiation of brain metastases is radiation necrosis, occurring in up to 25% of cases several months to years after irradiation [6].

Systemic treatments including targeted therapies, immunotherapy using checkpoint inhibitors, and antibody drug conjugates (ADCs) have also shown meaningful activity [7–10]. In combination with radiotherapy, these agents may increase the risk of radiation necrosis by approximately 5% [11]. Targeted therapies, particularly directed at epidermal growth factor receptor (EGFR) mutations or anaplastic lymphoma kinase (ALK) translocations in non-small-cell lung cancers, human epidermal growth factor receptor-2 (HER2)-positive breast cancers and BRAF gene (v-Raf murine sarcoma viral oncogene homolog B1) mutant melanoma are being used with increasing frequency in patients with brain metastases. Immune checkpoint inhibitors which have changed the therapeutic landscape of metastatic melanoma and lung cancers, are also increasingly used for brain metastases from these tumors. These agents are frequently used in combination with SRS, especially for patients with breast cancer brain metastases.

Magnetic resonance imaging (MRI), owing to its sensitivity and good spatial resolution, is the imaging modality of choice for diagnosis of brain metastases and guidance of therapeutic decisions. This is also the imaging of choice modality to evaluate treatment response in brain metastases following the response assessment (RANO) criteria [12]. However, as with primary brain tumors, differentiation of progressive disease from treatment-related effects using anatomical MRI can be challenging [6]. Contrast-enhanced MRI does not allow to accurately distinguish between brain metastases recurrence and treatment-related changes and more ‘advanced’ MRI techniques (e.g., diffusion weighted imaging and perfusion weighted imaging) can increase diagnostic accuracy [13]. However, diagnostic accuracy of conventional and advanced MRI remains suboptimal with reported sensitivities and specificities for advanced MR techniques around 80% [13], and thus there is ample room for improvement [14].

Positron-emission tomography (PET) is commonly used in patients with brain metastases, particularly in the post-treatment setting. Generally, regardless of the primary cancer type, the use of amino acid PET is recommended in addition to MRI to aid distinction between recurrent brain metastases and treatment-related changes [15, 16]. Although only one direct comparison study between the two types of radiotracers exists, amino acid PET is considered to outperform [^18^F]FDG PET in this regard [17]. The diagnostic accuracy of [^18^F]FDG for differentiating recurrent brain metastases from treatment-related changes varies significantly across studies [14, 18]. However, in situations where amino acid PET is not available, [^18^F]FDG may be useful.

The cellular uptake of amino acid radiotracers is mediated by L-amino acid transporters (LAT-1) [19], and has been shown to correlate with the presence of viable tumor cells in biopsy samples of brain metastases [20]. For the anti-1-amino-3-[F-18]-fluorocyclobutane-1-carboxylic acid, anti-3-[F-18] FACBC, [^18^F]fluciclovine, transport is mediated primarily by the neutral amino acid novel alanine serine cysteine transporters 2 (ASCT2), and to a lesser extent LAT-1, and has been demonstrated to have significantly higher image contrast (tumor-to-background) compared to methyl-[^11^C]-methionine ([^11^C]MET), owing to a limited penetration in the healthy brain [21].

A recent meta-analysis on the diagnostic performance of amino acid PET in distinguishing recurrent brain metastases from treatment-related changes, using radiotracers such as [^11^C]MET, O-(2-[^18^F]fluoroethyl)-L-tyrosine ([^18^F]FET), and 6-[^18^F]fluoro-L-dopa ([^18^F]FDOPA), reported pooled sensitivity and specificity of 82% and 84% [22]. Also, significant impact on the management of patients with a suspicion of brain metastasis recurrence based on amino acid PET results has been reported [23]. Studies suggest that amino acid PET provides better accuracy compared to contrast-enhanced MRI for the long-term follow-up of brain metastasis [24]. Cost-effectiveness of amino acid PET for distinguishing recurrent brain metastases from radiation-induced changes has been established in Germany [25, 26].

The utility of PET imaging for other purposes in brain metastases, such as initial diagnosis [27] or response assessment [28], needs to be further explored systematically, although it can be helpful in certain individual cases.

## Goals

The goals of this guideline are:

1) to assist nuclear medicine physicians in recommending, performing, interpreting and reporting the results of amino acids PET and [^18^F]FDG PET imaging in brain metastases,

2) to define procedure standards in clinical practice and clinical trials to harmonize data acquisition across centers for the application of amino acids PET and [^18^F]FDG PET imaging in brain metastases.

## Methods

The present guideline/procedure standard is based on the practice guidelines/procedures standards developed for glioma imaging published in 2019 [29] as well as the report of the PET/RANO group published in 2019 on PET imaging in brain metastases [14]. The present guideline/procedure standard will nevertheless focus on the technical aspects of amino acid PET and [^18^F]FDG PET imaging in brain metastases.

This guideline was brought to the attention of all other EANM Committees and to the National Societies of Nuclear Medicine, as well as to the EANO Guideline and the RANO Executive Committees.

## Indications and contraindications

### Indications

The most common indication for PET imaging in brain metastases is the differentiation between brain metastasis recurrence and treatment-related changes.

In general, amino acid PET imaging is achievable for brain metastases regardless of the site of cancer origin [19]. Use of amino acid PET in brain metastases of lung cancer, breast cancer and melanoma has been well documented. Evidence for other tumor types is lacking and needed, since there may be some variability in LAT-1 expression between tumor types. Lower LAT-1 expression in adenocarcinomas as compared with metastases from squamous cell carcinoma has been described, potentially leading to lower uptake, but evidence is limited [30]. Caution should also be made when evaluating metastasis of mucinous tumors as these may show low levels of uptake [31].

PET imaging of brain metastases is of limited value for characterization of small brain metastases (i.e. <10 mm in diameter, depending on the PET system spatial resolution) [27, 32, 33]. However, in case of contraindications for MRI and unclear CT findings, PET may be considered.

[^18^F]FDG PET is considered to have a lower diagnostic performance than amino acid PET in this setting [14, 17] due to the high physiological uptake of [^18^F]FDG in grey matter of the brain, reducing the contrast ratio between brain metastases and healthy brain [34].

However, imaging with [^18^F]FDG may be used in the absence of amino acid radiotracer availability, particularly if recurrence is suspected within a previously irradiated field (which could increase tumor-to-background ratio because of reduced uptake in surrounding brain), and for primary tumors that are known to show high [^18^F]FDG uptake. Brain metastases of primary tumors with mostly low [^18^F]FDG uptake (such as mucinous adenocarcinoma, lobular breast cancer, renal cancer, and so on) should not be evaluated with this tracer although a focal “cold spot” could also indicate presence of a large metastasis. It should be noticed that no significant association between the [^18^F]FDG uptake and degree of immune cell infiltration has been reported in brain metastasis [35].

Performing a brain acquisition systematically to search for unknown brain metastases in cancer patients receiving whole body [^18^F]FDG PET/CT is only of limited value [36–38]. Any suspicion of brain metastasis has to be explored by a brain MRI (or a CT in case of contra-indication).

For other indications, there are only very little data, but amino acid PET may be helpful for:

1. Primary diagnosis:

To identify regions with higher tumor cell content to guide biopsies [20] or radiotherapy planning [39].

2. Diagnosis of recurrence:

To guide radiotherapy planning [40].

3. Disease monitoring and response assessment:

To assess response to systemic therapy (for instance targeted therapy such as with immune checkpoint inhibitors), especially for differentiation between pseudoprogression and real progression following immunotherapy [28, 41, 42].

The clinical performance of PET imaging for each indication is discussed in a recent evidence-based recommendation by the PET/RANO group [14].

### Contraindications

Relative:

- Pregnancy
- Breast feeding if not discontinued. Of note: Discontinuation of breast feeding for 7 times the half-life (for fluorine-radiolabeled radiotracers 14 h) is considered sufficient.
- Patients who cannot remain lying down for at least 5 to 20 min.
- MRI contraindications if a PET/MR system is used (please refer to Sect. 1.3).

## Qualification and responsibilities of the personnel

- Physician:

PET scans must be conducted by a physician specialized in nuclear medicine or supervised by one, duly certified by accrediting boards. In Europe, the certified nuclear medicine physician overseeing the study and endorsing the report bears responsibility for the procedure, in accordance with national legislation and regulations. For guidelines in the United States, consult the SNMMI Guideline for General Imaging (https://s3.amazonaws.com/rdcms-snmmi/files/production/public/docs/General_Imaging_Version_6.0.pdf).

- Technologist:

PET scans should be carried out by competent registered/certified Nuclear Medicine Technologists. For detailed guidelines, please refer to the Performance Responsibility and Guidelines for Nuclear Medicine Technologists 3.1 and http://www.eanm.org/content-eanm/uploads/2016/11/EANM_2017_TC_Benchmark.pdf. In certain jurisdictions, additional qualifications may be required for technologists operating CT or MR components.

- Physicist:

PET scans should utilize PET systems that conform to national or international quality standards. A certified clinical physicist is responsible for ensuring compliance with these standards. Moreover, in specific countries, it is obligatory to have a board-certified medical physicist available to advise or aid personnel operating imaging systems and addressing any malfunctions. Additionally, scans must adhere to national or international dosimetry and radiation safety protocols to ensure the safety of both patients and staff.

### Prerequisites and patient preparation

#### PET imaging

- Appropriate clinical information about the patient and a clearly specified clinical question have to be defined to justify PET imaging.
- Information about the ability of the patient to cooperate for the examination and the participation of a caregiver can be helpful.
- History of prior therapy, including prior surgery, radiotherapy and systemic therapy, which might affect radiopharmaceutical distribution and toxicity should be reported. Particularly, a minimum delay of 1 month after the end of the radiotherapy and/or surgery for planning PET imaging is recommended to reduce the risk of false positive results due to inflammation processes.
- Results of pertinent resections and biopsies performed should also be collected.
- Recent morphologic imaging with MRI (including e.g. T1-weighted sequence before and after intravenous application of a gadolinium-based contrast agent, FLAIR and/or T2-weighted sequences, diffusion and perfusion weighted sequences) if available, must be retrieved.
- Information about recent epileptic seizures, as they can affect amino acid PET images by leading to false positive results [29].
- The patient should be informed by a physician or a technologist about the procedure to guarantee optimal compliance. Patient information and consent should be documented in the patient files according to local regulations.
- Body weight must be documented for Standard Uptake Value (SUV) measurements.
- The patient should be able to lie still for at least 5 to 20 min in case of static acquisitions (30 to 50 min if a dynamic amino acid PET is performed), depending on the sensitivity of the PET scan.
- Before scanning, the patients should empty their bladder for maximum comfort during the study and to reduce absorbed dose to bladder.
- Use of corticosteroids should be reported since they are known to potentially slightly decrease the uptake ratio by increasing healthy brain uptake of amino acid radiotracers, with dose/effect relationships needed to be further explored [43].
- The patient is requested to fast before at least 4 h the amino acid PET examination [44]. For consistency between scans, it is better to postpone the exam if the fasting is not well respected except in case of urgent indication.
- If sedation is required, it should start about 20–60 min before the scan in case of amino acid PET imaging, to avoid flux biases due to the sedation especially in case of dynamic imaging [29].
- No premedication is necessary, especially no Carbidopa is necessary prior to [^18^F]FDOPA imaging [45].

#### Specific considerations in case of [18F]FDG imaging

- The patient should be able to lie still for at least 5 to 20 min. In case of delayed acquisitions with [^18^F]FDG, which is performed in some centers, the patient should be informed to stay at least 3 to 5 h after the injection in the department (see 3.3 ‘PET imaging sequence’).
- Sedation should be administrated as late as possible after the radiotracer injection (typically at 30-minute post-injection), but before imaging.
- The patient is required to fast 6 h before PET imaging.
- Glycemia must be measured before PET imaging and in case of hyperglycemia (> 160 mg/dl; >8.9 mmol/L), the PET scan should be postponed [46]. Acute correction of hyperglycemia with insulin does not improve brain image quality because quantitation of regional cerebral glucose metabolism with [^18^F]FDG-PET requires steady-state situations and the normalization of an increased intracellular glucose level lags behind the normalization of the plasma glucose level [47].

#### PET/MR

All patients should be prescreened for potentially relevant MRI contraindications using a standardized checklist (e.g. previous contrast agent reactions, implants, ports, catheters, metallic implants, vascular stents, coils, active implants, cardiac pacemakers, bullets, claustrophobia, please see https://mrisafety.com/), if PET imaging is performed with PET/MRI.

- Any metallic items, such as dental prostheses, jewelry, and clothing with zippers or buttons, must be removed from the patient. The patient must be provided with cotton clothing. When it comes to implants, it’s essential to know the specific type, location, and material of the implant before scheduling an MRI examination. The patient’s implant pass must be requested and checked with the implant/device manufacturer, possibly online, to determine the implant’s safety level. There are three categories: “MRI unsafe” (an absolute contraindication), “MRI conditional” (a relative contraindication with specific conditions), and “MRI safe” (no contraindication).
- If the patient has implants, metal implants, or active devices labeled as “MRI conditional,” information (e.g., via the implant pass or online resources) must be obtained about all the conditions necessary for a safe MRI examination. Beyond safety considerations, implants can lead to artifacts, significant signal voids, and geometric distortions in MRI images, potentially complicating the interpretation of the images.

### Radiopharmaceuticals

The most commonly used PET tracers for brain metastasis imaging are:

Amino acid radiotracers:

[^18^F]FET: O-(2-[18F]fluoroethyl)-L-tyrosine.

[^18^F]FDOPA: 6-[18F]fluoro-L-dopa.

[^11^C]MET: methyl-[11 C]-methionine.

[^18^F]fluciclovine, previously known as [^18^F]FACBC: anti-1-amino-3-[18F]-fluorocyclobutane-1-carboxylic acid.

Glucose analogue radiotracer:

[^18^F]FDG: 2-deoxy-2-[18F]fluoro-D-glucose.

#### Preparation of the radiopharmaceuticals

Although there is currently no global regulatory framework for the manufacturing of radiopharmaceuticals, it should be carried out by qualified individuals, adhere to stringent quality control, and meet local regulatory standards [35, 36].

#### Administered activity

The injected activity should be adapted to national regulations and the used PET technology. The most commonly used activities for PET imaging in adults are currently as follows:

[^18^F]FET: 185–200 MBq.

[^18^F]FDOPA: 185–200 MBq.

[^11^C]MET: 370–555 MBq.

[^18^F]fluciclovine: 370 MBq [48, 49] but 185–200 MBq has been recently reported in brain metastases [50] or other primary brain tumors [51].

[^18^F]FDG: 185–200 MBq.

For children, injected activities should be reduced and adapted to the weight in compliance with the EANM Paediatric Dosage Card [52] or the North American Consensus Guidelines for Pediatric Administered Radiopharmaceutical Activities [53].

### PET acquisition protocols

Generally, image acquisition of the head is sufficient. During the entire investigation, continuous visual monitoring of the patient is necessary to account for motion. Monitoring is particularly important in patients with metastases-associated seizures.

For [^18^F]FDG PET/CT, a whole-body acquisition should be considered in individual patients for whom a systemic evaluation of neoplastic disease is required. Separate acquisition of the head in the same session is advised.

#### Positioning

Patients scan should be positioned in a dedicated head holder, with arms along the body. Extreme neck extension or flexion should be avoided. The entire brain should be in the field of view, including the entire cerebellum.

If a [^18^F]FDG whole-body scan is planned, image acquisition is performed in two separate steps: first whole-body, followed by the head to increase the contrast ratio between tumor and healthy brain uptake. The whole-body acquisition should be acquired with arms up to reduce artefacts in the torso.

#### Head stability

Patient should be informed immediately before PET acquisition to avoid head movements during all stages of the investigation. Head stability can be obtained by comfortably positioning the patient in the head holder, or other flexible head restraints, such as thermoplastic molds, vacuum mattresses, paddings and tape. Such restraints are frequently beneficial, particularly for the purpose of radiotherapy planning, and especially when dealing with children.

#### PET imaging sequence

Amino acid PET:

The preferred sequence of imaging is:

1) CT scout tomogram to establish the field of view and adjust where needed.

2) CT (low or high dose), MRI (attenuation correction sequence) or a 511 keV-transmission scan for attenuation correction.

3) Amino acid PET: Static single field of view (FOV) PET acquisition of the head for 10–20 min, 5 to 20 min post injection (p.i), depending on the radiotracer; the acquisition times may be shortened according to the scanner sensitivity.

3) Dynamic amino acid PET acquisition and generation of parametric images can provide additional information in search of brain metastasis recurrence [54–56].

3.3.2 In case of [^18^F]FDG: Static single FOV PET acquisition of the head for 10–20 min usually at 60 min p.i. for early acquisitions. To increase the tumor to healthy brain uptake ratio, traditionally delayed acquisitions are proposed (usually at 3–5 h p.i.) [57–59]. The PET/CT of the body can be acquired before the brain PET acquisition if needed (usually at 60 min p.i.). Again, the acquisition times may be shortened according to the scanner sensitivity.

#### Attenuation correction

Attenuation correction can be based on either a CT (low or high dose), MRI (attenuation correction sequence) or a 511 keV-transmission scan. When using a 511 keV-transmission scan, it’s crucial to acquire the transmission images before administering the tracer. CT parameters should always be selected to minimize patient radiation exposure while serving the intended purpose. For PET/MR, it is important to utilize a 3D data acquisition mode.

Several points related to attenuation correction in PET/MRI should be taken into consideration:

- Since attenuation correction relies on MRI data, the MRI must be accurate and free from artifacts to ensure precise PET quantification.
- Users should employ the most up-to-date MRI attenuation correction software, including ultrashort echo time (UTE), zero TE (ZTE) sequences, or bone models for bone detection in brain PET/MRI when available [60, 61].
- It is essential to be aware that MRI attenuation correction software may vary among different vendors and is regularly updated, often with performance changes that may not be well-documented.
- Different attenuation correction strategies for PET/MRI have been developed, and some may introduce systematic differences in the distribution of activity and calculated semi-quantitative metrics. These differences should be carefully considered during PET image interpretation [62, 63].
- MRI attenuation correction images should be routinely examined for artifacts, consistency, and plausibility during PET/MRI interpretation. Artifacts in MRI attenuation correction directly affect PET quantification in brain PET/MRI. Common artifacts include mis-segmentation of brain, fat, and bone tissue, as well as metal artifacts caused by dental prostheses and metallic implants such as coils, stents, surgical clips, and titanium calvarial implants/mesh [64–66]. These artifacts may manifest as signal voids that extend beyond the actual dimensions of metal inclusions, indicating regions of potentially inaccurate PET quantification [67].
- When applicable, consider using time-of-flight (TOF) PET detection to reduce the impact of metal artifacts in brain PET/MRI examinations [68].
- Ensure that radiofrequency head coils used are specifically labelled for combined PET/MRI. Using standard radiofrequency head coils designated for MRI only use will not be accounted for in PET/MRI attenuation correction and may result in inaccurate PET quantification and artifacts in PET images.

#### PET comparability

In case of longitudinal studies, the patient should be scanned, whenever possible, on the same system using the same procedures and PET tracer to avoid changes related to differences in imaging technology or methodology. To ensure PET comparability, a standardized protocol for image acquisition and clinical reading should be used. For further details, see also the section “PET image reconstruction” and “Documentation and reporting”. For prospective multicenter studies, the effective image resolution of brain PET scans should be harmonized to significantly reduce image quality variability while minimally affecting quantitative accuracy following the European recommendations [69].

Amino acid PET:

[^18^F]FET: 20 min static image acquisition obtained 20 min after injection. This may be part of a 40–50 min dynamic image acquisition initiated at tracer injection. In case of dynamic image acquisitions, they should be started using short frames that progressively increase in duration. From 10 to 50 min p.i, 5-min acquisition frames should be used to allow the tracer uptake slope to be assessed during this interval. To allow precise information on the tracer uptake phase to be obtained, the following image acquisition frame sequences could be used during the first 10 min after injection: 12 frames of 5 s, 6 frames of 10 s, 6 frames of 30 s and 5 frames of 60 s.

[^18^F]FDOPA: 10–20 min static image acquisition obtained 10–20 min after injection. This may be part of a 30–40 min dynamic image acquisition initiated at the time of tracer injection. Dynamic image acquisitions should be started using frames that progressively increase in duration like for [^18^F]FET or be based on 30 frames of 1 min each [70].

[^11^C]MET: 20 min static image acquisition obtained 10 min after injection. This may be part of a 35 min dynamic image acquisition initiated at tracer injection which can be recorded using frames that progressively increase in duration like for [^18^F]FET or as follows: 14 time frames (time frames 1–5, 1 min; 6–8, 2 min; 9–11, 3 min; 12–14, 5 min) [71].

[^18^F]fluciclovine: 10 min static image acquisition obtained at least 10 min p.i. The time activity curve plateaus after 10 to 20 min [51, 72].

Movement artefacts: If movement artefacts are expected, it may be helpful to acquire the static time window dynamically, e.g., in 5-min frames, or in list mode. The sinograms must be checked and only those of the properly acquired motion-free time period for reconstruction must be used.

In case of [^18^F]FDG PET imaging: 10–20 min static image acquisition usually obtained at 60 min after injection (early phase images). It may be useful to plan a second series of static [^18^F]FDG PET images at approximately 3 to 5 h after injection (delayed phase images) to increase the tumor to healthy brain uptake ratio [57–59]. Glucose loading before [^18^F]FDG PET imaging has also been proposed to increase the tumor to healthy brain ratio, but this approach is currently less used [73, 74].

### PET image reconstruction

During the process of image reconstruction, it is essential to apply all the necessary corrections for accurate quantitative assessment. This includes corrections for attenuation, scatter, random events, dead time, and decay, along with detector sensitivity normalization. While Time-of-Flight (TOF) acquisitions and reconstructions are permissible, their specific benefits for brain imaging are still being thoroughly investigated [75].

The current standard in the field is iterative reconstruction, which should be the preferred approach. Additionally, the use of resolution modeling during reconstruction, often referred to as point-spread-function (PSF) reconstructions, has the potential to enhance the sharpness of brain metastasis delineation and improve detectability in brain tumors [76]. However, it’s important to note that this approach can introduce Gibbs artifacts [77] and may lead to quantitative errors that depend on tumor size and the model of the PET/CT scanner in use.

To standardize PET image quality, particularly in multi-center scenarios, EARL guidelines for IQ recovery have been established even if dedicated guidelines to brain oncological PET imaging are expected [78]. In most cases, using a higher resolution reconstruction can enhance the ability to visually interpret images and delineate tumors. If a particular PET system permits the use of various reconstruction settings, it is advisable to employ a dedicated high-resolution reconstruction protocol for brain imaging. The voxel size for PET reconstruction should fall within the range of 1 to 2 mm, with a reconstructed spatial resolution of less than 6 mm full width at half maximum.

### Interpretation / quantification

#### General image display

For optimal value range, PET images should ideally employ a minimum of 16-bit pixels, and for effective image presentation, appropriate image scaling is essential. Consideration can be given to employing a color scale. When showcasing PET images, it is advisable to present them in the transaxial orientation, and they should also be compared with morphological images in the coronal and sagittal planes. Utilizing internal landmarks for reorientation can help achieve a standardized image display, with reorientation procedures often relying on the intercommissural line [79].

Amino acid PET: [^18^F]FET, [^11^C]MET, [^18^F]FDOPA: the color scale should be adjusted so that the background radioactivity of the healthy brain is in the lower third of the range. For [^18^F]FDOPA, the striatum uptake can be adjusted with the maximal of the color scale. For [^18^F]fluciclovine, the maximum of the color scale should be adjusted to the lesion uptake.

In case of [^18^F]FDG PET imaging: the color scale should be adjusted so that the healthy brain uptake is the maximal of the color scale. If the lesion uptake is higher than the healthy brain uptake, the maximum of the color scale should be adjusted to the lesion uptake.

### Image analysis

The image analysis is based on the combination of both visual and semi-quantitative analyses. In the context of treatment-related changes vs. recurrence, it is essential to provide a semi-quantitative evaluation, together with the description of the pattern of uptake since interpretation based on visual analysis only can be misleading as there is usually a mixture of radiation-induced necrosis and brain metastasis recurrence. Image analysis should help the clinicians to identify the presence of recurrent tumor in the context of treatment-related changes.

Calculation of the standardized uptake value (SUV) may be performed by dividing the radioactivity concentration (kBq/ml) in the tissue by the radioactivity (MBq) injected per body weight (kg), body surface area (m^2^) or lean body mass (kg) (depending on the most appropriate distribution volume for each tracer). However, since tumor-to-background ratios (TBR) have proven reliable markers in previous neuro-oncology studies, calculation of SUV is not mandatory (except for [^18^F]fluciclovine where SUV exhibits higher performances values than TBR) [48, 49]).

Standard summation images described in the chapter PET comparability are used for clinical reading and evaluated together with fusion to recent MRI sequences, at least contrasted-enhanced T1-weighted and T2/FLAIR-weighted sequences. For PET/MR co-registration, the vendor-provided co-registration software packages are sufficient but still require visual verification. For amino acid PET, co-registration should be done using the scalp and the nose as references.

Amino acid PET.

In a first visual analysis, a subjective qualitative evaluation can be performed, but should be systematically associated to the semi-quantitative analysis.

[^18^F]FET, [^11^C]MET, [^18^F]FDOPA: the lesion of interest can be classified as either *positive*, when tracer uptake exceeds the background activity or as *negative*, when no or only low uptake can be found. For [^18^F]fluciclovine, due to very low physiological uptake in the brain, defining a lesion as *negative* based on background comparison can be misleading [51], and other reference tissue than healthy brain can be used, e.g. the parotid gland [50]. For [^18^F]FDOPA, a visual ratio between the lesion and the striatum can be used to evaluate *positivity* [80]. In case of *positive* lesion, the nuclear medicine physician can additionally determine if the uptake is homogeneous or heterogeneous. In case of heterogeneous uptake, the nuclear medicine physician can indicate areas of intense tumoral uptake, in relation to MR characteristics on fused images.

In a second step, a semi-quantitative analysis is performed, based on uptake ratios.

[^18^F]FET, [^11^C]MET, [^18^F]FDOPA: the TBR is calculated by dividing the activity measured within the lesion (SUV_max_ for TBR_max_ or SUV_mean_ for TBR_mean_) by the SUV_mean_ of the contralateral healthy brain. The reference of the contralateral healthy brain is obtained by delineating a large crescent-shaped area gathering grey- and white-matter tissues at the level of the centrum semi-ovale as recommended [81]. The calculation of the TBR_mean_ needs a reproducible method for the segmentation step of the biological tumor volume to avoid inter- and intra-readers variability (please see 5.3.1). It is also possible to use a maximum lesion to maximum background uptake ratio, calculated by dividing the SUV_max_ of the lesion by the SUV_max_ of the healthy brain, however, it is considered that this method is more sensitive to noise. For [^18^F]FDOPA, a tumor to striatum ratio (TSR) can be calculated. The definition of the striatum volume of interest may be based using a threshold of 70% of the SUV_max_ striatum [^18^F]FDOPA uptake and corrected manually when required [82].

[^18^F]fluciclovine: Measures of SUV, in particular SUV_max_, provide better performance results than measures of TBR, due to the very low uptake of normal healthy brain [48, 49].

Amino acid PET dynamic analyses: dynamic analyses can help in the interpretation of amino acid PET imaging. A visual analysis can be first performed to appreciate the type of time-activity curve (TAC): constantly increasing, presenting a plateau phase, or decreasing. Secondly, semi-quantitative parameters can be obtained from the TAC: time to peak, i.e. the time to reach the maximal activity for the lesion and slope during the second part of the TAC (typically after 10 min). For extracting the TAC, a volume of interest of 2 mL should be defined regards the lesion, either by determining SUV_peak_ within the metabolic tumor volume, with a fixed diameter of 1.6 cm centered on the maximal lesion uptake, or within regions of interest within a 90% isocontour defined slice by slice in early summation images (10–30 min p.i.). In brain metastases, only dynamic analyses have been reported for [^18^F]FET in literature [54–56], but dynamic analyses are also feasible with [^18^F]FDOPA PET [83], [^11^C]MET [71] and [^18^F]fluciclovine [51].

In case of [^18^F]FDG PET imaging:

Co-registration with MRI is easier than for amino acid PET since the normal healthy brain exhibits physiological uptake.

Visual analysis: the lesion of interest can be classified as either *positive*, when tumor uptake is at least equal to the background activity, the latter being measured in either the contralateral grey-matter (more specific), contralateral white-matter (more sensitive), or combined [29]. When tumor uptake is lower compared to the background activity, lesions can be considered as *negative*. When feasible, delayed acquisitions (3 to 5 h p.i.) are more favorable than early ones (45 min–1 h p.i.) to enhance the lesion to background ratio [58]. The difference in uptake between delayed and early acquisitions can also be used, when available [57]. If the lesion is *positive*, the characteristics of the uptake (homogeneous or heterogeneous) should be described in relation to the MR images.

Semi-quantitative analysis: TBR is calculated by dividing the activity measured within the lesion (SUV_max_ for TBR_max_ or SUV_mean_ for TBR_mean_) by the SUV_max_ or SUV_mean_ of the contralateral healthy brain. The reference of the contralateral healthy brain is obtained by doing a mirror volume of interest than those of the lesion encompassing grey- and/or white-matter. The volume of interest of the lesion is calculated by means of fusion to MR images (preferentially the T1 weighted contrast-enhanced sequence). It is important to apply the same semi-quantitative analysis between early and delayed acquisitions images to calculate the percentage of wash out from the lesion.

#### Cut-off thresholds for definition of biological tumor volume

Amino acid PET:

[^18^F]FET: TBR of 1.6 [84].

[^11^C]MET: TBR of 1.3 [85].

[^18^F]FDOPA: TBR of 2.0 [86].

[^18^F]fluciclovine: SUV of 4.8 based on preliminary data [50].

The proposed thresholds for [^18^F]FET, [^11^C]MET and [^18^F]FDOPA have been determined through a population of primary/recurrent glioma lesions [84–86], with no histological proven studies available in brain metastases. In gliomas, the threshold of 1.6 for TBR has been chosen for [^18^F]FET, [^11^C]MET and [^18^F]FDOPA for consistency in the PET-based response assessment criteria for diffuse gliomas (PET RANO 1.0) [87].

[^18^F]FDG: not available.

#### Interpretation of PET data

For the main indication of the differential diagnosis between brain metastasis recurrence of treatment-related changes:

Amino acid PET.

Table 1 summarizes the methodology employed and the main interpretation criteria used for differentiating brain metastases recurrence from treatment-related changes with amino acid PET imaging.

[^18^F]FET, [^11^C]MET, [^18^F]FDOPA and [^18^F]fuciclovine: Only for [^18^F]FDOPA, a visual analysis based on the striatum uptake has been proposed [80]. However, for amino acid PET imaging, a semi-quantitative analysis is performed with calculation of TBR. Details of TBR cut-offs proposed in the search of recurrence for each amino acid PET radiotracer are available in Table 1. It is advised to use TBR values based on the SUV_mean_ and not the SUV_max_ of the reference region, as it is less sensitive to noise. In any case, the same methodology should be applied regards PET acquisitions of brain metastases in a same center or across centers for data harmonization in multicenter studies. It should be noted that for [^18^F]fluciclovine, SUV measurements provide better diagnostic performances than TBR, due to the very low uptake in healthy brain tissue [48, 49].

Similar to what is currently done with MRI, performing repeated amino acid PET imaging can be useful for scans with ambiguous results (i.e. values close to the cut-off values). Follow-up amino acid PET scan should be planned after the initial PET scan to evaluate the changes of the TBR (which typically clearly increases in case of brain metastases recurrence and is stable or decreases in case of treatment related changes) [24]. Of note, follow-up scans should be acquired with the same PET tracer used at initial examination. PET imaging should ideally be planned in parallel with brain MRI, every 2–3 months depending on the therapeutic management of the patient or when clinically indicated.

Dynamic analyses can be interpreted in adjunct to static ones, to aid the differential diagnosis. Visually, a constantly increasing TAC is in favor of treatment-related changes while other types of TAC are in favor of brain metastases recurrences. Semi-quantitatively, early TTP (i.e. before 20 min) or plateau (i.e. close to 0) or negative slopes are more in favor of brain metastases recurrence.

**Table 1:** Thresholds associated to the best diagnostic performances for discriminating brain metastases recurrences from treatment-related changes in studies focusing only on patients with brain metastases scanned with amino acid PET. Thresholds presented are from analog PET scanners

PET Tracer	Method	Threshold for brain metastasis recurrence	Refs
[^11^C]MET	TBR	TBR_mean_ ≥ 1.42	[88]
[^11^C]MET	TBR	Maximum lesion to maximum background uptake ratio ≥ 1.61	[89]
[^11^C]MET	TBR	Maximum lesion to maximum background uptake ratio ≥ 1.40	[90]
[^11^C]MET	TBR	Maximum lesion to maximum background uptake ratio ≥ 1.42	[17]
[^11^C]MET	SUV, TBR	SUV_max_ ≥ 3.29 TBR_max_ ≥ 2.03 TBR_mean_ ≥ 1.33	[91]
[^18^F]FDOPA	Visual	Lesion ≥ striatum	[80]
[^18^F]FDOPA	TBR	Maximum lesion to maximum background uptake ratio ≥ 1.59	[92]
[^18^F]FDOPA	TBR	Maximum lesion to maximum background uptake ratio ≥ 1.92	[24]*
[^18^F]FET	TBR + dynamic	Association of TBR_mean_ ≥ 1.9 and curve with early TTP (≤ 20 min)	[54]^‡^
[^18^F]FET	TBR + dynamic	Association of TBR_mean_ ≥ 1.95 and curve with a slope ≤ 0.37 SUV/hour	[55]
[^18^F]FET	TBR + dynamic	Association of TBR_max_ ≥ 2.15/TBR_mean_ ≥ 1.95 and decreasing time activity curve	[56]
[^18^F]fluciclovine	SUV, TBR, dynamic	SUV_max_ ≥ 4.3	[49]

* obtained from serial PET acquisitions, ^‡^partial common population with [55]

Although diagnostic performances are lower than those of amino acid PET, Table 2 summarizes the methodology employed and the main interpretation criteria used for differentiating brain metastases recurrences from treatment-related changes with [^18^F]FDG PET imaging.

[^18^F]FDG: A visual analysis is recommended by comparing lesion uptake to those of the healthy brain (preferentially the grey-matter area, associated with more specificity), and can be sufficient [58, 93]. If semi-quantitative analysis is performed, measurement of the maximum lesion to background uptake ratio is preferred. Delayed images (i.e. 3 to 5 h p.i.) are preferred over early images for the interpretation of lesion uptake, to increase the lesion to background uptake ratio [58]. An increase of 19% for the lesion/grey matter SUV_max_ ratio between early and delayed images has been reported as efficient to identify brain metastases recurrence [57].

**Table 2:** Thresholds associated to the best diagnostic performances for discriminating brain metastases recurrences from treatment-related changes in studies focusing only on patients with brain metastases scanned with [^18^F]FDG PET. Thresholds presented are from analog PET scanners

PET Tracer	Method	Threshold for brain metastasis recurrence	Refs
[^18^F]FDG	Visual	Lesion > grey matter	[93]
[^18^F]FDG	Visual	Lesion uptake	[94]
[^18^F]FDG	Semi quantitatively (60 min and ≈ 3 h post injection)	An increase of 19% of the lesion/grey matter SUV_max_ ratio between early and delayed images	[57]
[^18^F]FDG	SUV_max_	SUV_max_ ≥ 3	[95]
[^18^F]FDG	Visual, TBR	Lesion > contralateral grey matter on delayed images (4–5 h post injection)	[58]

For other indications:

Response assessment:

One study investigated treatment monitoring of immunotherapy and targeted therapy using [^18^F]FET in patients with melanoma and lung cancer brain metastases [28]. Metabolic responders to immunotherapy and targeted therapy on [^18^F]FET PET had a significantly longer stable follow-up (threshold of TBR reduction of follow-up scans relative to baseline, ≥ 10% with an accuracy of 82%).

No comparable data are available for other amino acid PET radiotracers or [^18^F]FDG. PET-based response criteria similar to those proposed for gliomas are not available so far for brain metastases [87].

At primary diagnosis:

Due to the low spatial resolution of PET, PET imaging is of limited value at the initial diagnosis of brain metastases, especially if their maximal diameter is inferior to the spatial resolution of the current PET systems (resolution i.e. ≤ 5 mm).

One study investigated the value of [^18^F]FET PET in patients with newly diagnosed and untreated brain metastasis, reporting that [^18^F]FET uptake was highly variable across brain metastases and not linked to the origin of cancer. The highest variability of uptake was observed in melanoma metastases [27].

#### Physiological tracer distribution

Use of [^18^F]FET, [^11^C]MET, [^18^F]FDOPA and [^18^F]fluciclovine results in physiological uptake in vascular structures, such as venous sinuses, basal ganglia, cerebellum, skin, bone marrow of the skull, and lacrimal- and salivary glands. Occasionally, slight uptake in the pineal gland, choroid plexus and clivus bone marrow has also been described. For [^18^F]FDOPA, moderate to high uptake in basal ganglia related to the physiological distribution of the radiotracer is usual, and some uptake can be observed in the raphe nuclei [96]. For [^18^F]fluciclovine, these physiological uptakes are less observed related to a better lesion to background ratio [48, 49].

[^18^F]FDG PET:

Physiological uptake of the healthy brain is predominantly observed in grey-matter areas. Physiological uptake in the extraocular muscles can also be reported. These physiological uptake can be reduced by using delayed PET acquisitions [57, 58].

#### Known pitfalls

All tracers:

Treatment-related changes are often associated with inflammatory processes, which can induce false positive results in amino acid PET and [^18^F]FDG PET imaging. Therefore, a minimum delay of 1 month between the end of radiotherapy and/or surgery and PET imaging is recommended, to reduce the risk of false positive results.

When applying thresholds for differentiation, they should preferentially advantage the specificity rather than the sensitivity.

Uptake may be decreased in lesion for which the size is below the spatial resolution of the PET system used (i.e., especially those close to or below 10 mm in diameter).

As a semi-quantitative analysis is recommended, a bolus delivery of the radiotracer is assumed to be consistent with serial SUV measures or dynamic analyses. Therefore, potential infiltration or extravasation of administered dose during injection could negatively affect these calculations.

Specific pitfalls in amino acid PET and [^18^F]FDG PET radiotracers have been previously reported [29, 97].

## Documentation and reporting

The description of findings in brain tumor imaging should generally comply with guidelines as published previously for brain tumor imaging with radiolabeled amino acids and [^18^F]FDG, for [^18^F]FDG imaging in oncology, and with regard to general aspects of reporting, such as due diligence [29, 78].

The content of the report affects patient management and is a legal document. It is good practice to provide a structured report with concise concluding statements intended to answer the specific clinical question(s) posed, if possible.

Reports should contain the following general structure:

General information:

- Name of the patient and other identifiers, such as birthdate.
- Name of the referring physician.
- Type and date of examination.
- Name of radiotracer including route of administration and amount of activity administered.
- Patient history with emphasis on diagnosis and brain metastases related treatment (particularly recent radiotherapy/surgery) and clinical question leading to study request.
- Known allergies and hypersensitivities.
- Documentation of patient information and consent according to local regulations.

Body of the report:

Procedure description:

- Information on the imaging procedure (e.g. static or dynamic acquisitions, PET, PET/CT, PET/MRI), contrast media and interval between PET tracer injection and image acquisition.
- If [^18^F]FDG is used, report also the measure of the glycemia before injection and the dual phase imaging if performed.
- If sedation is performed, describe type and time of medication in relation to the tracer injection.

Data quality:

- Abnormal tracer biodistribution.
- CT- or MRI- related attenuation artifacts e.g., from metallic implants.
- Any observed events that may adversely influence interpretation, e.g. head movements, seizure activity.
- Poor compliance for fasting.
- Steroids medications.

Comparative data:

- PET/CT images should be co-registered and compared to MRI.
- PET/CT images should be compared to previous PET/CT scans to evaluate the course of disease.
- The type and date of comparative data should be noted before the description of imaging findings.

Description of findings:

- Both pathological uptake and physiological uptake (briefly) should be described: e.g., normal versus abnormal radiotracer uptake in brain, head and neck area.
- In case of abnormal findings, an anatomically correct description of the location(s), the extent(s) (e.g. infiltrated structures) and the intensity of pathological tracer accumulation(s) related to normal tissue uptake should be described.

The uptake characteristics include:

- Patterns of uptake e.g., focal, diffuse, ring-shaped, homo- vs. inhomogeneous.
- Intensity of uptake of the lesion(s) of interest: visually slight, moderate or strong uptake.
- Extent and peak correlated to morphological imaging, e.g., abnormalities on diagnostic CT, low-dose CT or MRI (T1 contrast enhancement and/or T2/FLAIR hyperintensity).

Semiquantitative parameters:

- Report the TBR_max_ and TBR_mean_ for each lesion of interest (or SUV in case of [^18^F]fluciclovine); report of the biological tumor volume is optional. Importantly, keep the same methodology to extract the semiquantitative parameters across different reports in a same center.
- For dynamic analysis, if performed, the type of TAC should be described. The extraction of TTP and slope are optional.
- Comparison to previously performed PET studies, e.g., in case of ambiguous PET scans results and necessity to perform a follow-up scan in the differential diagnosis between brain metastases recurrence and treatment-related changes.
- Clinically relevant incidental findings should be reported, e.g. extracerebral metastases if a whole-body PET is performed.

Limitations:

When appropriate, factors that limit data quality or diagnostic accuracy should be mentioned.

Conclusion/interpretation:

The interpretation, integrating both the visual and semi-quantitative analyses should address the question raised in the clinical request and integrate medical history, comparative imaging and any limitations. A precise diagnosis should be given whenever possible. In the context of the differential diagnosis between brain metastases recurrences and treatment-related changes, as a mixture of radiation induced necrosis and recurrence is often visualized, additional scans or follow-up scans should be recommended when appropriate.

## Equipment specifications

### System specifications

It is advised to utilize state-of-the-art 3D PET/CT or PET/MRI systems. These systems should allow for the acquisition of (low dose) CT images or MRI-based sequences suitable for attenuating and correcting scatter in the PET emission data. Alternatively, dedicated brain PET-only systems may be employed, provided they are equipped with transmission scan sources of adequate strength, as recommended by the vendor, to ensure high-quality transmission scans and, consequently, effective attenuation correction of the PET emission data. PET(/CT) systems should feature a minimum axial field of view of 15 cm to ensure comprehensive coverage of the entire brain, encompassing the cerebellum and brain stem.

### PET acquisition

The system must possess the capability to acquire PET emission data in 3D mode. Data can be reconstructed either online or offline (i.e., retrospectively) in single or multiple frames. Additionally, PET images can be reconstructed with or without attenuation correction. While non-attenuation-corrected PET images are not primarily used for interpretation, they can be valuable for identifying attenuation artifacts in the attenuation-corrected PET images (e.g., in case of metal artifacts). The system should incorporate all necessary functionalities and methods required for quantitative brain PET imaging and reconstruction. These include, but are not limited to, online randoms correction, scatter correction, attenuation correction, dead time correction, decay and abundance correction, and normalization (correction for detector sensitivities).

## Quality control and improvement

The quality and quantitative aspects of PET images can be influenced by a range of factors [98]. To ensure that PET images maintain a high level of quality, quantitative accuracy, and consistency, which is especially crucial for comparing PET images in multi-center studies, it is essential to routinely conduct various quality control (QC) experiments on PET systems. Detailed descriptions of these QC experiments have been previously provided [29].

## Radiation safety

The systematic use of radiotracers leads to systemic exposure of the patients to radiation. The radiation dose from low-dose CT scans of the head region depends on the CT scanning parameters and is generally well below 0.5 mSv. The overall effective dose from PET/CT investigations of the head region, when accounting for the whole-body exposure, should remain near or below 5 mSv. Details of radiation dosimetry for [^18^F]FET, [^18^F]FDOPA and [^11^C]MET and [^18^F]FDG, have already been reported in previous guidelines for PET imaging in gliomas (please see Table 3 of the reference [29].

For [^18^F]fluciclovine, the mean effective dose when accounting for the whole-body exposure is 0.0221 mSv/ MBq [99].

## Conclusion

This joint practice guideline/procedure standard, collaboratively developed by the EANM, the SNMMI, the EANO and the PET/RANO group, supplement those on PET imaging in gliomas [29] since they are focused on the most frequent brain tumors, brain metastases.

The aim of this guideline is to assist nuclear medicine physicians in recommending, performing, interpreting and reporting the results of brain PET imaging in patients with brain metastases. This practice guideline will define procedure standards for the application of PET imaging in patients with brain metastases in routine practice and clinical trials and will help to harmonize data acquisition and interpretation across centers.

## Data Availability

Not applicable.

## Declarations

### Approval

This practice guideline was approved by the Board of Directors of the EANM, SNMMI, EANO and PET/RANO Working Group.
